# Prediction of acute kidney injury in patients with femoral neck fracture utilizing machine learning

**DOI:** 10.3389/fsurg.2022.928750

**Published:** 2022-07-26

**Authors:** Jun Liu, Lingxiao Xu, Enzhao Zhu, Chunxia Han, Zisheng Ai

**Affiliations:** Department of Medical Statistics, Tongji University School of Medicine, Shanghai, China

**Keywords:** femoral neck fracture, acute kidney injury, postoperative, machine learning, model interpretation, MIMIC-IV database

## Abstract

**Background:**

Acute kidney injury (AKI) is a common complication associated with significant morbidity and mortality in high-energy trauma patients. Given the poor efficacy of interventions after AKI development, it is important to predict AKI before its diagnosis. Therefore, this study aimed to develop models using machine learning algorithms to predict the risk of AKI in patients with femoral neck fractures.

**Methods:**

We developed machine-learning models using the Medical Information Mart from Intensive Care (MIMIC)-IV database. AKI was predicted using 10 predictive models in three-time windows, 24, 48, and 72 h. Three optimal models were selected according to the accuracy and area under the receiver operating characteristic curve (AUROC), and the hyperparameters were adjusted using a random search algorithm. The Shapley additive explanation (SHAP) analysis was used to determine the impact and importance of each feature on the prediction. Compact models were developed using important features chosen based on their SHAP values and clinical availability. Finally, we evaluated the models using metrics such as accuracy, precision, AUROC, recall, F1 scores, and kappa values on the test set after hyperparameter tuning.

**Results:**

A total of 1,596 patients in MIMIC-IV were included in the final cohort, and 402 (25%) patients developed AKI after surgery. The light gradient boosting machine (LightGBM) model showed the best overall performance for predicting AKI before 24, 48, and 72 h. AUROCs were 0.929, 0.862, and 0.904. The SHAP value was used to interpret the prediction models. Renal function markers and perioperative blood transfusions are the most critical features for predicting AKI. In compact models, LightGBM still performs the best. AUROCs were 0.930, 0.859, and 0.901.

**Conclusions:**

In our analysis, we discovered that LightGBM had the best metrics among all algorithms used. Our study identified the LightGBM as a solid first-choice algorithm for early AKI prediction in patients after femoral neck fracture surgery.

## Introduction

Hip fracture, a major public health problem in the aging population (1) causing a tremendous clinical and economic burden on healthcare services (2, 3). Acute kidney injury (AKI), also known as acute renal failure, is a common postoperative complication in patients undergoing hip surgery. It is related to increased risk of morbidity and mortality, as well as a longer hospital stay, and higher medical costs (4, 5).

The previously reported incidence of AKI after hip fracture surgery ranges from 8% to 24%, owing to postoperative care conditions and the definition of AKI (6–8). Patients with AKI have a higher risk of postoperative complications, such as infection, transfusion, and death (9). One study on the national incidence and outcomes of AKI in patients undergoing total hip arthroplasties (THA) showed that patients with AKI have a 7.52-fold increased risk of death. Even a minor increase in creatinine levels after THA is associated with a greater increase in healthcare utilization (10).

As there are no effective treatments for AKI, early identification and management are critical. Identifying patients at high risk of AKI prior to diagnosis appears to have better outcomes than treating only diagnosed AKI (11).

However, early identification of AKI remains challenging, as AKI is defined by increased creatinine or decreased urine output, both of which are late, nonspecific indicators of the underlying disease (12). Although partial models have been developed to identify patients at high risk of AKI (13–16), these models rely heavily on intensive care unit (ICU) data, which is unlikely to be available at the time of admission (17). Typically, model performance improves during ICU admission; however, many patients with hip fractures are not admitted to the ICU. In addition, most risk models are built using logistic regression, which requires statistical assumptions regarding the linear relationship between variables and outcomes. Excluding features based on the aforementioned criteria can result in a significant loss of information and omission of unanticipated associations that could be used to increase predictive power.

Machine learning is now widely used in medicine to develop predictive models for a large number of features and complex nonlinear relationships. Because many postoperative patients with AKI are not admitted to the ICU, this study aimed to develop predictive tools to predict the risk of AKI in patients with femoral neck fractures based solely on hospitalization data.

## Materials and methods

### Source of data

We enrolled a cohort of patients with femoral neck fractures from the Medical Information Mart from Intensive Care (MIMIC)-IV version 1.0. MIMIC-IV (18), built upon the success of MIMIC-III, is a real-world and publicly available clinical database maintained by the Beth Israel Deaconess Medical Center from 2008 to 2019 (19). The web-based course offered by the National Institutes of Health was completed, and certification (researcher certificate number: 9848944) was obtained.

### Selection of participants

**Figure 1:**
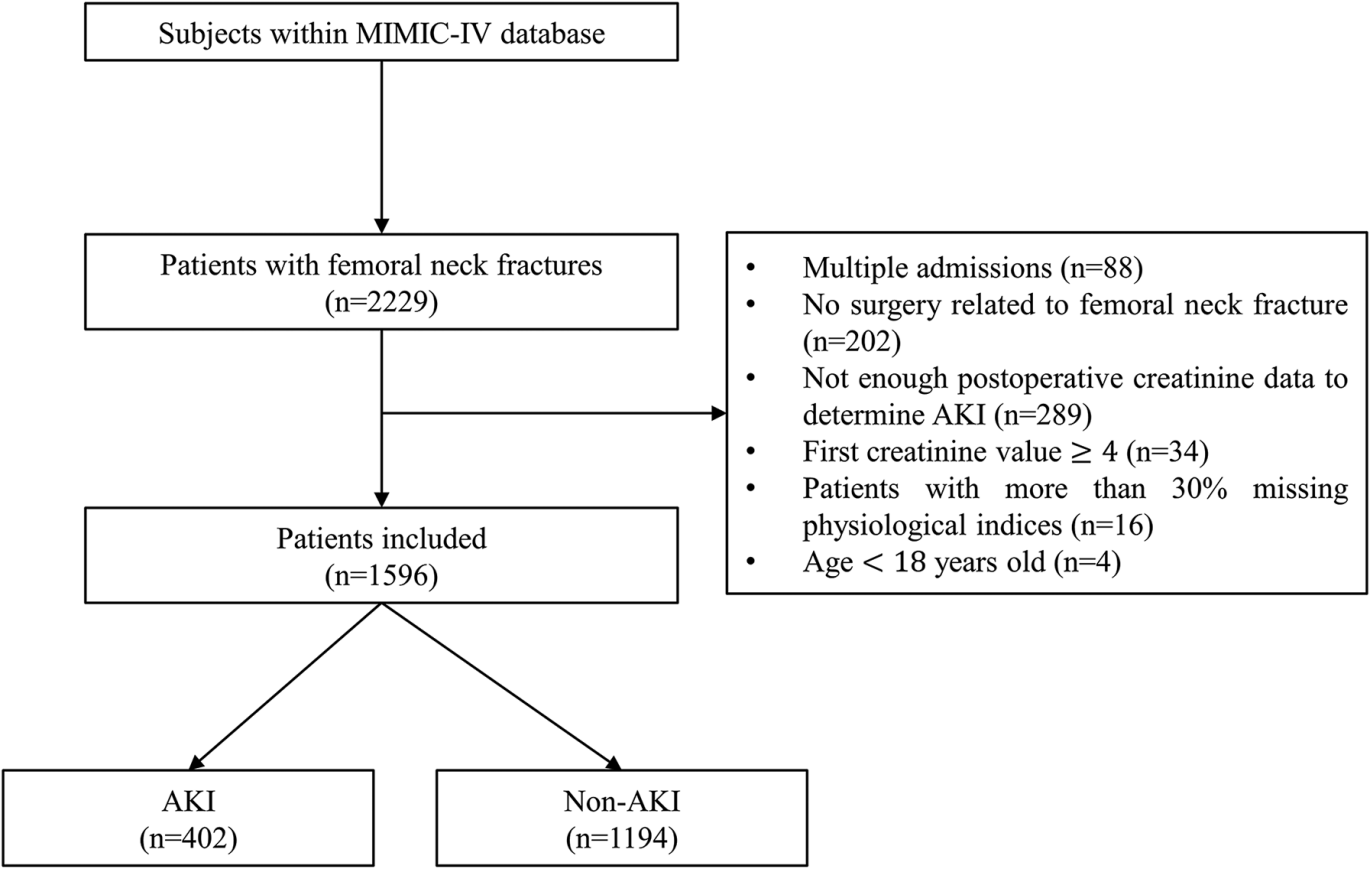
Flow chart of patient selection.

Patients diagnosed with femoral neck fractures were included according to the International Classification of Diseases (ICD) version 9. The inclusion criteria were age ≥18 years, experiencing a hip fracture for the first time, and undergoing hip fracture surgery. Patients without sufficient serum creatinine data to determine the occurrence of AKI were excluded. To rule out patients with severe kidney problems, patients whose initial serum creatinine ≥4.0 mg/dl were excluded based on risk, injury, failure, loss, end-stage kidney disease (RIFLE) and acute kidney injury network (AKIN) criteria (20, 21). The flow chart of patient selection is shown in Figure 1.

### Outcome (AKI)

According to the Kidney Disease: Improving Global Outcomes (KDIGO) criteria (22), AKI following femoral neck fracture surgery was defined during the first 7 days after the operation. Urine output criteria for AKI were not included because of the scarcity of urine output data from general hospital wards in the MIMIC database. Postoperative AKI was defined as either an increase in serum creatinine ≥0.3 mg/dl within 48 h or a greater than 1.5-fold increase at baseline. The lowest measured creatinine level during the previous 7 days was defined as the baseline creatinine value. Our primary outcome was AKI within 24 h, and secondary outcomes were AKI within 48 and 72 h.

### Predictors of AKI

Demographic characteristics, physiological indicators, comorbidities, and relevant interventions are usually considered predictor variables. Based on the KDIGO criteria and literature (23, 24), the following variables were collected from the general ward: patient demographics [age, sex, ethnicity, and marital status] and laboratory index [anion gap (mmol/L), bicarbonate (mEq/L), blood urea nitrogen (mEq/L), calcium (mg/dl), chloride (mEq/L), creatinine (mg/dl), glucose (mg/dl), hemoglobin (g/dl), mean corpuscular volume (fL), platelet count (10^9^/L), red blood cell count (10^12^/L), red blood cell distribution width (%), white blood cell count (10^9^/L), potassium (mEq/L), and sodium (mEq/L)]. In addition, comorbidities based on the recorded ICD versions 9 and 10 were collected, including chronic kidney disease, myocardial infarction, congestive heart failure, liver disease, chronic obstructive pulmonary disease, hypertension, diabetes mellitus, dementia, and cancer. Lastly, medications known to affect renal function include diuretics, nephrotoxic antibiotics, non-steroidal anti-inflammatory drugs (NSAIDs), angiotensin-converting enzyme inhibitors (ACEI), red blood cell transfusion (RBCT), and mechanical ventilation (MV). We also calculated the maximum, minimum, and mean values of each physiological characteristic prior to the development of AKI, which were treated as separate variables in the final dataset.

### Data collection windows

**Figure 2:**
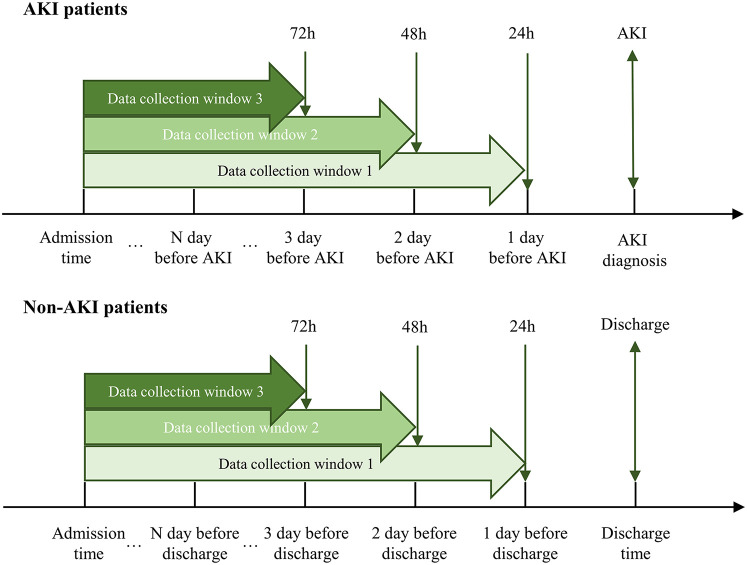
Data collection windows.

This study considered three-time windows, 24, 48, and 72 h, for AKI prediction. As shown in Figure 2, the data collection window for patients with AKI was between the day of admission and 24, 48, or 72 h before AKI diagnosis. The data collection window for patients with non-AKI was between the date of admission and 24, 48, or 72 h before discharge or death.

### Statistical analysis

We used data extraction from the MIMIC-IV using BigQuery. The baseline characteristics of the AKI and non-AKI groups were compared. Continuous variables were reported as the mean and standard deviation (if normal) or as the median and interquartile range (if non-normal). Categorical variables were presented as numbers and percentages (%). For comparisons of continuous variables, the t-test or Mann-Whitney U test was used, and for the comparison of categorical variables, the chi-square test or Fisher’s exact test was used, as appropriate.

The imputation method was not used because the advanced boosting machine learning method can handle missing values automatically; in contrast, when training other models, the missing values of continuous variables were imputed using median values, and categorical variables were imputed using mode values. First, the prediction performance of the 10 machine learning algorithms was compared using 10-fold cross-validation using the PyCaret Python package (version 2.3.6). Next, we calculated the area under the receiver operating characteristic curve (AUROC) and the accuracy for each fold to evaluate the performance of the various models. We then selected the top three performing models and used the random search algorithm to tune the hyperparameters of the models. Compared with grid search, random search is faster and more suitable for larger hyperparametric search space, which using the Scikit-Learn Python package (version 0.23.2). In this study, the best performing optimization model was the final AKI prediction model, defined as the full model. The Shapley additive explanation (SHAP) analysis was used to evaluate the positive or negative effects of the relevant features, which using a game theoretic approach to estimate the importance of each feature in the validation set (25). Based on the SHAP values, we selected important features and retrained a more compact model, which may be more useful in clinical circumstances.

Statistical Package for the Social Sciences (SPSS) (version 26.0) was used for comparison of baseline characteristics, and *P* < 0.05 was considered statistically significant. The models were developed using Python (version 3.8.5).

## Results

### Baseline characteristics

As shown in Supplementary Table S1, for all 2,229 patients with femoral neck fracture in the MIMIC-IV, 1,596 were enrolled in the final cohort. During their hospital stay, 402 patients developed AKI, while 1,194 did not. Patients with AKI were more likely to be older (median age, 83.00 years vs. 80.00 years; *P* = 0.001), have longer ICU stay time (2.26 days vs. 1.82 days; *P* = 0.013), have a high laboratory value, and have more comorbidities. In addition, patients who develop AKI are more likely to require medical intervention and treatment, such as blood transfusion and mechanical ventilation.

### Comparison of 10 models

The data collected in each time window were separated into training (70%) and validation sets (30%). Then, the training set from each time window was used to construct models using 10 machine-learning algorithms and to make preliminary comparisons. The predictive performance of each model is presented in Table 1. As shown, for the 24 h prediction, the performance of the logistic regression was acceptable (accuracy: 0.864; AUROC: 0.870). The ensemble algorithms outperformed others in terms of accuracy and AUROC, such as light gradient boosting (LightGBM) (accuracy: 0.904; AUROC: 0.924), extreme gradient boosting (XGBoost) (accuracy: 0.891; AUROC: 0.920), and gradient boosting decision tree (GBDT) (accuracy: 0.887; AUROC: 0.913). As the prediction time window increases, the performance of each model decreases. According to the 48 h prediction models, the risk of AKI could be predicted with an accuracy of 0.815 to 0.887 and an AUROC of 0.681 to 0.858. According to the 72 h prediction models, the risk of AKI could be predicted with an accuracy of 0.831 to 0.931 and an AUROC of 0.717 to 0.885. The 48 and 72 h prediction models increase the accuracy, but this comes at the cost of lowering the AUROC value. For each time window, we selected the three best models and optimized them in the following stage.

**Table 1:** Internal validation performance of various models.

Models	24 h		48 h		72 h	
	Accuracy	AUC	Accuracy	AUC	Accuracy	AUC
LightGBM	**0** **.** **904**	**0**.**924**	0.886	0.857	0.927	0.819
XGBoost	0.891	0.920	**0**.**887**	0.855	0.923	0.828
GBDT	0.887	0.913	0.882	0.857	**0**.**931**	0.837
Ada boost Classifier	0.876	0.894	0.861	0.810	0.906	0.788
Random Forest	0.872	0.907	0.876	**0**.**858**	0.926	0.857
LDA	0.858	0.862	0.873	0.836	0.912	0.866
Logistic Regression	0.864	0.870	0.870	0.831	0.897	0.833
Extra Trees Classifier	0.863	0.900	0.877	0.855	0.922	**0**.**885**
Naive Bayes	0.822	0.824	0.833	0.781	0.831	0.816
Decision Tree	0.788	0.718	0.815	0.681	0.894	0.717

LightGBM, light gradient boosting machine; XGBoost, extreme gradient boosting; GBDT, gradient boosting decision tree; LDA, linear discriminant analysis; AUC, area under receiver operating characteristic curve.

The bold values represent the model that perform best in each time window.

**Figure 3:**
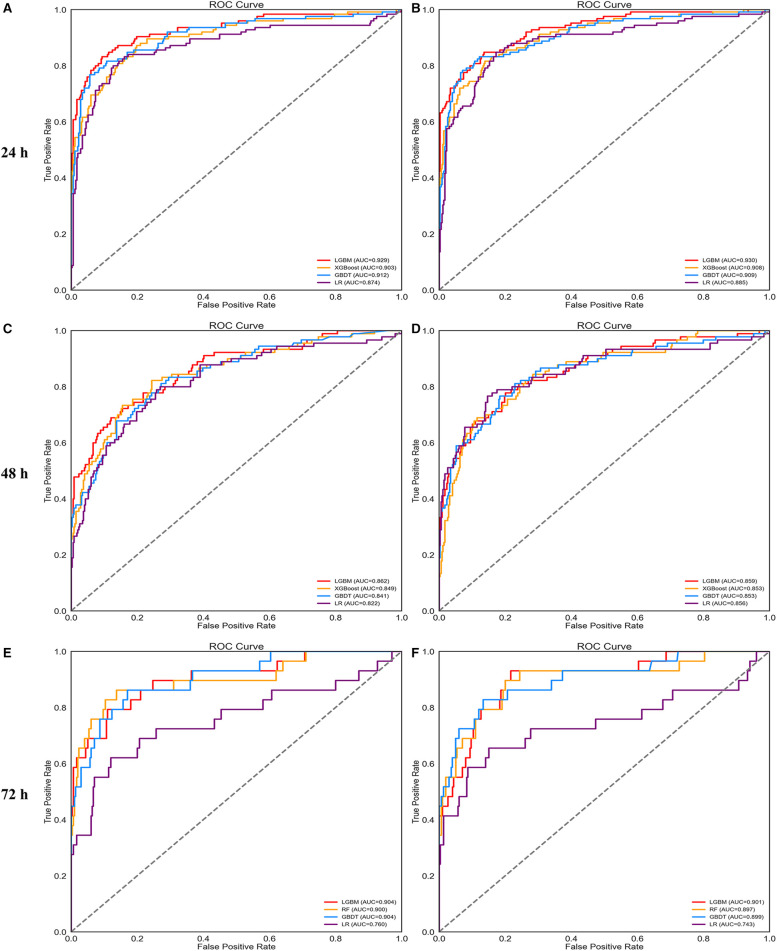
ROC curves of four prediction models using all features (**A,C,E**) and important features (**B,D,F**).

For 24 and 48 h predictions, the hyperparameters of the LightGBM, XGBoost, and GBDT models were adjusted. For 72 h prediction, random forest, GBDT, and LightGBM models were selected and optimized in the next step. Table 2 lists the performance of the models in the validation set after tuning each time window. The LightGBM model had the most powerful discrimination for AKI prediction in all time windows. The ROC curves of each model after parameter adjustment are shown in Figure 3. In addition, the ROC curve of the logistic regression was added to facilitate comparison.

**Table 2:** Performance of full models in the validation set.

	Full model	Accuracy	AUC	Recall	Prec.	F1	Kappa
24 h	Light Gradient Boosting Machine	0.898	0.929	0.680	0.904	0.776	0.712
	Extreme Gradient Boosting	0.875	0.903	0.696	0.798	0.744	0.661
	Gradient Boosting Decision Tree	0.894	0.912	0.688	0.878	0.771	0.703
	Logistic Regression	0.858	0.874	0.656	0.766	0.707	0.614
48 h	Light Gradient Boosting Machine	0.875	0.862	0.478	0.843	0.610	0.542
	Extreme Gradient Boosting	0.857	0.849	0.444	0.755	0.559	0.481
	Gradient Boosting Decision Tree	0.854	0.841	0.422	0.760	0.543	0.464
	Logistic Regression	0.843	0.822	0.467	0.667	0.549	0.457
72 h	Light Gradient Boosting Machine	0.945	0.904	0.448	0.867	0.591	0.565
	Random Forest Classifier	0.942	0.900	0.345	1.000	0.513	0.490
	Gradient Boosting Decision Tree	0.942	0.904	0.448	0.813	0.578	0.549
	Logistic Regression	0.921	0.761	0.345	0.588	0.435	0.395

### Compact models

Supplementary Figure S1 plots the bar chart of the SHAP values for each model, which sorts features by the mean of the SHAP value. The figure shows that the top 20 features of different prediction models at 24, 48, and 72 h affected the output of the models strongly. Thus, we summarized the important features given by the 9 models, deleted the repeated features, and constructed the important feature data set (58 features). Next, the compact models were built based on these selected features. Figures 3B,D,F shows the ROC curves for each compact model. Finally, Table 3 shows the performance of the compact models in the validation set. The performance of the compact models was similar to those of the full model but considered to be more practical in clinical practice.

**Table 3:** Performance of compact models in the validation set.

	Compact model	Accuracy	AUC	Recall	Prec.	F1	Kappa
24 h	Light Gradient Boosting Machine	0.896	0.930	0.672	0.903	0.771	0.705
	Extreme Gradient Boosting	0.875	0.909	0.664	0.822	0.735	0.654
	Gradient Boosting Decision Tree	0.889	0.909	0.704	0.846	0.769	0.697
	Logistic Regression	0.858	0.885	0.640	0.777	0.702	0.610
48 h	Light Gradient Boosting Machine	0.870	0.859	0.433	0.867	0.578	0.511
	Extreme Gradient Boosting	0.859	0.853	0.611	0.671	0.640	0.552
	Gradient Boosting Decision Tree	0.868	0.853	0.511	0.767	0.613	0.538
	Logistic Regression	0.872	0.856	0.511	0.793	0.622	0.549
72 h	Light Gradient Boosting Machine	0.948	0.901	0.414	1.000	0.585	0.563
	Random Forest Classifier	0.945	0.897	0.448	0.867	0.591	0.565
	Gradient Boosting Decision Tree	0.933	0.899	0.517	0.652	0.577	0.541
	Logistic Regression	0.930	0.743	0.414	0.667	0.511	0.475

### Model interpretation

**Figure 4:**
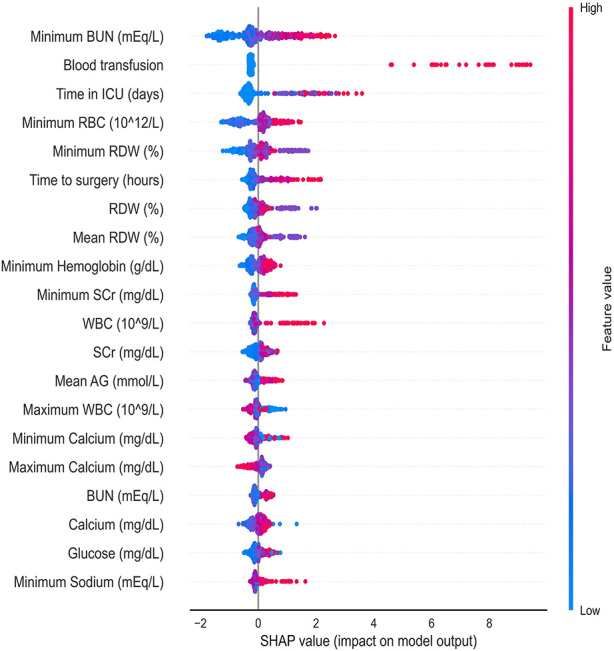
Distribution of the impact that each feature has on the full 24 h prediction model output estimated using the SHapley Additive exPlanations (SHAP) values. The plot sorts features by the sum of SHAP value magnitudes over all samples. The color represents the feature value (red high, blue low). The x axis measures the impact on the model output (right positive, left negative). BUN, blood urea nitrogen; ICU, intensive care unit; RBC, red blood cell; RDW, red blood cell distribution width; SCr, serum creatinine, WBC, white blood cell count; AG, anion gap.

Figure 4 shows a SHAP summary plot of the LightGBM on the full model output to reveal the distribution of the effects of each feature. Every row in the figure indicates a feature; the horizontal coordinate represents the SHAP value, and a point represents a sample. The redder the color, the greater the value of the feature itself. The minimum BUN value before AKI was important for model prediction. The higher the minimum BUN level, the more likely the patient would develop AKI after surgery. Postoperative blood transfusions also showed the importance of model prediction. In most patients without postoperative transfusion, the SHAP values are concentrated around 0; however, in patients with blood transfusion, the SHAP values are much higher than 0, showing a positive influence. Other laboratory values related to renal function and metabolism, such as RBC, RDW, hemoglobin, WBC, glucose, and sodium, displayed strong clinical predictive power.

**Figure 5:**
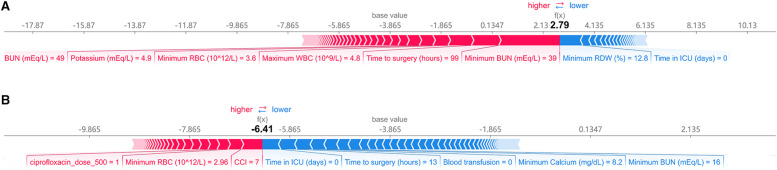
Explanation of the prediction results for specific instances. The base value (−3.865) is the average value of the predictive model; the output values are the predicted AKI risks. The bars in red and blue represent risk factors and protective factors, respectively; longer bars mean greater feature importance. Here, these values are the model outputs before the SoftMax layer, and therefore, they are not equal to the final predicted probabilities. This figure shows the explanation for a high-risk instance (**A**) and a low-risk instance (**B**). BUN, blood urea nitrogen; RBC, red blood cell count; WBC, white blood cell count; RDW, red cell distribution width; CCI, charlson comorbidity index; ICU, intensive care unit.

Figure 5 shows the predicted results for two concrete examples. The risk and protective factors are shown as red and blue bars, respectively. The longer bars indicate important features. Figure 5A shows an example of a high-risk patient. Although the patient was not admitted to the ICU, she had high levels of BUN, WBC, and potassium values and had a long waiting time before surgery. The model correctly predicted that a patient would develop AKI. In Figure 5B, the patient did not receive a blood transfusion and was not admitted to the ICU. The patient’s condition was mild, our model predicted that he would be less likely to develop AKI.

## Discussion

AKI has been reported to occur in 15%–40% of high-energy trauma patients (25–28). A few published studies have found that AKI occurs in 15%–24% of patients with hip fractures (4, 7, 29). In this study, the prevalence of AKI was similar to that reported in previous studies (25%). We also found that patients with postoperative AKI had higher in-hospital mortality (7.5% vs. 0.7%; *P* < 0.001) and longer hospital stay (162 days vs. 115 days; *P* < 0.001) than those without AKI. As there is no effective treatment available for AKI, early recognition and management are critical. Although several models have been developed for the early AKI prediction, they are mostly used for emergency patients. Currently, there is no accurate AKI predictive tool for patients undergoing orthopedic surgery.

In this study, various machine learning models for early AKI prediction were developed, which were used to predict AKI in three-time windows: 24, 48, and 72 h prior to AKI detection. Accuracy, AUROC, recall, precision, F1 score, and kappa value were used to evaluate each model's performance. The results showed that logistic regression had the worst performance, whereas the XGBoost and GBDT algorithms displayed satisfactory performance. In general, the LightGBM showed the best results. In addition, LightGBM was found to perform best in AKI prediction among the models constructed from important features. Even 3 days before the onset of AKI, the compact model constructed with LightGBM could accurately predict AKI development (accuracy: 0.948; AUROC: 0.901). The proposed models provide early AKI prediction from 1 to 3 days, allowing for prompt intervention in patients at high risk of AKI, thereby improving patient outcomes.

In our study, we discovered that gradient boosting models outperformed other algorithms in predicting AKI. In brief, Gradient boosting, a powerful machine learning technique, aims to increase the emphasis on observations that are poorly modeled by a set of existing base learners by repeatedly training them (30). LightGBM is an efficient and scalable implementation of tree-based gradient-boosting approaches for machine learning. A histogram-based algorithm is used in the LightGBM to reduce memory utilization and speed up training. In addition, it uses a leaf wise split strategy, rather than a level wise split strategy to build significantly more complicated trees, which is the major element in obtaining greater accuracy. However, this can lead to overfitting, which can be prevented by increasing the maximum depth option. It is distributed and effective, with the following benefits: faster running speed, lower memory usage, higher efficiency, improved accuracy, large-scale data processing capabilities, and support for parallel and GPU learning, as well as direct input categorical features (without one-hot coding) (31).

In general, the more valuable the variables, the better the model will discriminate, but the clinical usability will be worse. Therefore, in this study, two models were developed. The full models were developed based on 86 clinical variables, and the highest AUROC was obtained. However, gathering 86 clinical features and applying them to the full models is difficult. Therefore, using the complete model in hospitals with modern electronic health record systems is recommended. Therefore, compact models based on important features were developed to be suitable for most clinical situations, which have similar performance to the full model but are easier to apply in clinical practice.

The SHAP values were also used for the interpretability of the models. As shown in Figure 4, positive effects were seen for most of the top features, which implies that the higher the feature value, the greater the likelihood of developing AKI. In this study, renal function indicators (BUN) were the most important predictors of AKI, followed by perioperative blood transfusion. The SHAP value was very high in patients who received blood transfusions, indicating a higher risk of AKI. In addition, the length of ICU stay, preoperative waiting time, and laboratory test results (such as RBC count and RDW) can help predict imminent AKI. Furthermore, as shown in Figure 5, the prediction results are also presented at the individual level, which enables our model to visually analyze individual risk factors.

Our study had several limitations. First, AKI was determined based only on serum creatinine levels according to the KDIGO criteria. Urinary output, an indication of AKI, was not included because general ward hospitalization did not contain urine volume data. Previous studies have reported that the absence of urine output data prevents a more precise AKI definition (32, 33), while urinary output may be a more sensitive marker than serum creatinine for early AKI detection (34). Second, we only analyzed data from a single center with a relatively small number of participants. There were not enough cases (32 patients developed stage II AKI or higher after surgery) to develop robust models for different stages of AKI. In terms of model evaluation, although we adopted a 10-fold cross-validation for model evaluation, it is imperative to validate the models externally to prevent overfitting for large datasets with various patient characteristics and standards of care. Third, the majority of features were extracted manually from the MIMIC-IV database, we are constructing an automated electronic health record system that can gather patient data from various sources in real-time. Using these techniques, the prediction models based on machine learning algorithms could be useful in clinical practice. Finally, since the models are trained based on the input features, which not take into account ICU-generated or overlooked features, some hidden relationships may be missed. Future prospective studies are required to construct models for different stages of AKI and evaluate the use of predictive models in clinical settings.

## Conclusions

In conclusion, based on machine-learning algorithms, we successfully developed a predictive tool for postoperative AKI prediction in patients with femoral neck fractures within 72 h. The proposed models used demographics, physiological indicators, comorbidities, medications, and relevant interventions to detect AKI earlier than serum creatinine levels alone. We also performed the SHAP analysis to assess the positive and negative effects of important features on AKI prediction, which improved model's predictability. The development of software to optimize the treatment for patients with femoral neck fractures is ongoing, with the goal of reducing the risk of AKI following surgery.

## Data Availability

Publicly available datasets were analyzed in this study. This data can be found here: The datasets are available in the physionet (https://physionet.org/content/ mimiciv/0.4/).
