# Supplementary material for: Prediction of acute kidney injury in patients with femoral neck fracture utilizing machine learning
Source: Front Surg. 2022 Jul 26;9:928750. doi: 10.3389/fsurg.2022.928750 (PMC9360500; doi:10.3389/fsurg.2022.928750)
Supplement: Supplementary file 1 [file Table_1_v1.docx]

Supplementary Material

# Supplementary Table

**Table S1 |** Comparisons of features between the AKI and the non-AKI

| Variables | AKI  (*n* = 402) | Non-AKI  (*n* = 1194) | *P* |
| --- | --- | --- | --- |
| **Demographics** |  |  |  |
| Age | 83.00 [72.00, 88.00] | 80.00 [66.00, 87.00] | 0.001 |
| Gender |  |  |  |
| Male (%) | 151 (37.6%) | 370 (31.0%) | 0.015 |
| Female (%) | 251 (62.4%) | 824 (69.0%) |  |
| Ethnicity |  |  |  |
| Caucasian | 350 (87.1%) | 1035 (86.7%) | 0.845 |
| Other | 52 (12.9%) | 159 (13.3%) |  |
| Time in Hosp, days | 162.00 [115.00, 252.00] | 115.00 [90.00, 156.25] | $<$0.001 |
| ICU | 143 (35.6%) | 160 (13.4%) | $<$0.001 |
| Time in ICU, days | 2.26 [1.10, 5.83] | 1.82 [0.91, 3.26] | 0.013 |
| In-hospital mortality | 30 (7.5%) | 8 (0.7%) | $<$0.001 |
| **Interventions** |  |  |  |
| Blood transfusion | 63 (15.7%) | 0 (0%) | $<$0.001 |
| Mechanical Ventilation | 102 (25.4%) | 138 (11.6%) | $<$0.001 |
| Time to surgery (hours) | 32.00 [24.00, 54.00) | 28.00 [23.00, 42.00] | <0.001 |
| Surgery type |  |  |  |
| Fixation | 287 (71.4%) | 893 (74.8%) | 0.180 |
| Replacement | 115 (28.6%) | 301 (25.2%) |  |
| **Laboratory parameters** |  |  |  |
| Anion gap_first (mmol/L) | 13.96 (3.05) | 13.01 (2.59) | $<$0.001 |
| Anion gap_max (mmol/L) | 15.21 (3.00) | 14.44 (2.43) | $<$0.001 |
| Anion gap_min (mmol/L) | 12.42 (3.14) | 10.47 (2.10) | $<$0.001 |
| Anion gap_mean (mmol/L) | 13.76 (2.66) | 12.37 (1.83) | $<$0.001 |
| Bicarbonate_first (mEq/L) | 25.36 (4.23) | 25.62 (3.18) | 0.261 |
| Bicarbonate_max (mEq/L) | 26.61 (4.41) | 28.13 (3.07) | $<$0.001 |
| Bicarbonate_min (mEq/L) | 23.54 (4.36) | 23.98 (3.03) | 0.06 |
| Bicarbonate_mean (mEq/L) | 25.09 (3.96) | 26.08 (2.70) | $<$0.001 |
| BUN_first (mEq/L) | 25.00 [18.00, 35.25] | 17.00 [12.00, 22.00] | $<$0.001 |
| BUN_max (mEq/L) | 28.00 [20.00, 40.00] | 18.00 [14.00, 25.00] | $<$0.001 |
| BUN_min (mEq/L) | 22.00 [16.00, 32.00] | 13.00 [9.00, 18.00] | $<$0.001 |
| BUN_mean (mEq/L) | 15.41 [11.50, 21.00] | 25.00 [18.00, 35.13] | $<$0.001 |
| Calcium_first (mg/dL) | 8.52 (0.68) | 8.41 (0.55) | 0.007 |
| Calcium_max (mg/dL) | 8.66 (0.62) | 8.61 (0.51) | 0.178 |
| Calcium_min (mg/dL) | 8.15 (0.80) | 8.00 (0.53) | 0.001 |
| Calcium_mean (mg/dL) | 8.40 (0.63) | 8.30 (0.45) | 0.004 |
| Chloride_first (mEq/L) | 103.43 (4.98) | 103.45 (4.11) | 0.934 |
| Chloride_max (mEq/L) | 105.29 (5.36) | 105.41 (4.12) | 0.686 |
| Chloride_min (mEq/L) | 101.81 (5.37) | 100.74 (4.07) | $<$0.001 |
| Chloride_mean (mEq/L) | 103.58 (4.78) | 103.05 (3.75) | 0.046 |
| Creatinine_first (mg/dL) | 1.20 [0.80, 1.60] | 0.80 [0.60, 1.00] | $<$0.001 |
| Creatinine_max (mg/dL) | 1.20 [0.90, 1.80] | 0.80 [0.70, 1.10] | $<$0.001 |
| Creatinine_min (mg/dL) | 1.10 [0.70, 1.50] | 0.70 [0.50, 0.90] | $<$0.001 |
| Creatinine_mean (mg/dL) | 1.20 [0.80, 1.65] | 0.75 [0.60, 0.97] | $<$0.001 |
| Glucose_first (mg/dL) | 128.50 [107.75, 160.00] | 126.00 [109.00, 147.00] | 0.221 |
| Glucose_max (mg/dL) | 150.50 [127.00, 188.25] | 146.00 [127.00, 171.25] | 0.076 |
| Glucose_min (mg/dL) | 111.50 [94.00, 136.00] | 103.00 [92.00, 117.25] | $<$0.001 |
| Glucose_mean (mg/dL) | 124.00 [111.42, 139.50] | 133.00 [113.30,158.63] | $<$0.001 |
| Hemoglobin_first (g/dL) | 10.39 (1.69) | 10.75 (1.66) | $<$0.001 |
| Hemoglobin_max (g/dL) | 10.76 (1.57) | 11.23 (1.37) | $<$0.001 |
| Hemoglobin_min (g/dL) | 9.35 (1.69) | 8.96 (1.42) | $<$0.001 |
| Hemoglobin_mean (g/dL) | 10.04 (1.45) | 10.04 (1.21) | 0.919 |
| MCV_first (fL) | 92.14 (7.36) | 91.51 (6.40) | 0.121 |
| MCV_max (fL) | 93.25 (7.38) | 92.77 (6.26) | 0.244 |
| MCV_min (fL) | 91.11 (7.18) | 89.72 (5.83) | $<$0.001 |
| MCV_mean (fL) | 92.11 (7.06) | 91.20 (5.91) | 0.020 |
| Platelet_first (10^9^/L) | 192.00 [154.00, 247.25] | 204.00 [159.00, 257.00] | 0.009 |
| Platelet_max (10^9^/L) | 205.00 [163.00, 275.00] | 236.00 [188.00, 310.00] | $<$0.001 |
| Platelet_min (10^9^/L) | 172.00 [127.75, 220.00] | 170.00 [134.00, 213.00] | 0.962 |
| Platelet_mean (10^9^/L) | 189.60 [148.88, 245.06] | 201.50 [160.42, 253.47] | 0.002 |
| Potassiu_first (mEq/L) | 4.34 (0.65) | 4.12 (0.51) | $<$0.001 |
| Potassiu_max (mEq/L) | 4.62 (0.75) | 4.42 (0.50) | $<$0.001 |
| Potassiu_min (mEq/L) | 4.07 (0.68) | 3.72 (0.39) | $<$0.001 |
| Potassiu_mean (mEq/L) | 4.33 (0.61) | 4.05 (0.34) | $<$0.001 |
| RBC_first (10^12^/L) | 3.44 (0.59) | 3.54 (0.58) | 0.003 |
| RBC_max (10^12^/L) | 3.56 (0.56) | 3.70 (0.50) | $<$0.001 |
| RBC_min (10^12^/L) | 3.09 (0.60) | 2.95 (0.50) | $<$0.001 |
| RBC_mean (10^12^/L) | 3.32 (0.52) | 3.31 (0.44) | 0.563 |
| RDW_first (%) | 14.50 [13.60, 15.70] | 13.90 [13.20, 14.80] | $<$0.001 |
| RDW_max (%) | 14.90 [13.90, 16.10] | 14.60 [13.70, 15.80] | $<$0.001 |
| RDW_min (%) | 14.40 [13.50, 15.60] | 13.70 [13.00, 14.60] | $<$0.001 |
| RDW_mean (%) | 14.68 [13.75, 15.90] | 14.16 [13.42, 15.20] | $<$0.001 |
| Sodium_first (mEq/L) | 138.38 (4.11) | 137.90 (3.60) | 0.038 |
| Sodium_max (mEq/L) | 139.62 (4.33) | 139.56 (3.39) | 0.793 |
| Sodium_mean (mEq/L) | 136.31 (5.39) | 135.20 (3.61) | $<$0.001 |
| Sodium_mean (mEq/L) | 138.06 (4.05) | 137.40 (3.13) | 0.003 |
| WBC_first (10^9^/L) | 9.60 [7.30, 12.60] | 9.60 [7.50, 12.23] | 0.925 |
| WBC_max (10^9^/L) | 11.20 [8.60, 15.10] | 11.30 [9.20, 14.20] | 0.953 |
| WBC_min (10^9^/L) | 8.40 [6.40, 11.20] | 7.50 [6.10, 9.10] | $<$0.001 |
| WBC_mean (10^9^/L) | 9.84 [7.69, 12.64] | 9.23 [7.70, 11.21] | $<$0.001 |
| **Comorbidities** |  |  |  |
| Hypertension | 189 (47.0%) | 625 (52.3%) | 0.064 |
| CKD | 137 (34.1%) | 127 (10.6%) | $<$0.001 |
| Diabetes | 132 (32.8%) | 224 (18.8%) | $<$0.001 |
| CHF | 149 (37.1%) | 169 (14.2%) | $<$0.001 |
| MI | 62 (15.4%) | 87 (7.3%) | $<$0.001 |
| CCI | 6.37 (2.36) | 4.80 (2.26) | $<$0.001 |
| **Medication** |  |  |  |
| Diuretics | 190 (47.3%) | 416 (34.8%) | $<$0.001 |
| NSAIDs | 135 (33.6%) | 348 (29.1%) | 0.094 |
| ACEI | 89 (22.1%) | 216 (18.1%) | 0.074 |
| Nephrotoxic antibiotics | 86 (21.4%) | 177 (14.8%) | 0.002 |

ICU, intensive care unit; Hosp, hospital; BUN, blood urea nitrogen; MCV, mean corpuscular volume; RBC, red blood cell; RDW, red blood cell distribution width; WBC, white blood cell; CKD, chronic kidney disease; CHF, congestive heart failure; MI, myocardial infarction; CCI, charlson comorbidity index; NSAIDS, non-steroidal anti-inflammatory drugs; ACEI, angiotensin-converting enzyme inhibitors.

# Supplementary Figure


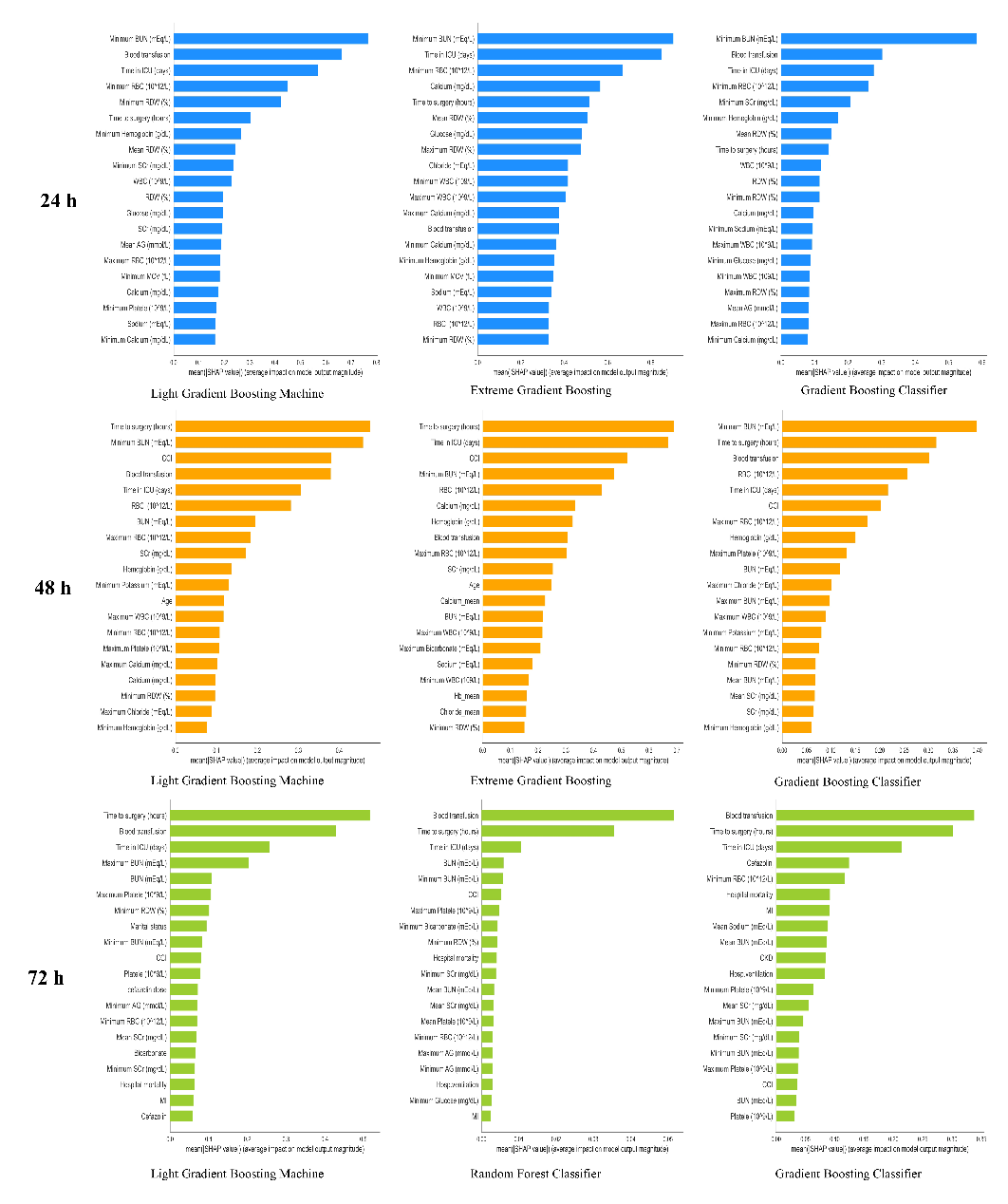


**Supplementary Figure 1** | Top 20 features with high mean SHAP values of the three best models in each time window
